# Mutation landscape in patients with myelofibrosis receiving ruxolitinib or hydroxyurea

**DOI:** 10.1038/s41408-018-0152-x

**Published:** 2018-11-22

**Authors:** Annalisa Pacilli, Giada Rotunno, Carmela Mannarelli, Tiziana Fanelli, Alessandro Pancrazzi, Elisa Contini, Francesco Mannelli, Francesca Gesullo, Niccolò Bartalucci, Giuditta Corbizi Fattori, Chiara Paoli, Alessandro M. Vannucchi, Paola Guglielmelli

**Affiliations:** 1 CRIMM, Centro di Ricerca e Innovazione per le Malattie Mieloproliferative, Azienda Ospedaliera Universitaria Careggi, Dipartimento di Medicina Sperimentale e Clinica, Università degli Studi, Firenze, Italy; 2 Doctorate School GenOMeC, University of Siena, Siena, Italy

## Abstract

Refractoriness to ruxolitinib in patients with myelofibrosis (MF) was associated with clonal evolution; however, whether genetic instability is promoted by ruxolitinib remains unsettled. We evaluated the mutation landscape in 71 MF patients receiving ruxolitinib (*n* = 46) and hydroxyurea (*n* = 25) and correlated with response. A spleen volume response (SVR) was obtained in 57% and 12%, respectively. Highly heterogenous patterns of mutation acquisition/loss and/or changes of variant allele frequency (VAF) were observed in the 2 patient groups without remarkable differences. In patients receiving ruxolitinib, driver mutation type and high-molecular risk profile (HMR) at baseline did not impact on response rate, while HMR and sole *ASXL1* mutations predicted for SVR loss at 3 years. In patients with SVR, a decrease of ≥ 20% of *JAK2*V617F VAF predicted for SVR duration. VAF increase of non-driver mutations and clonal progression at follow-up correlated with SVR loss and treatment discontinuation, and clonal progression also predicted for shorter survival. These data indicate that (i) ruxolitinib does not appreciably promote clonal evolution compared with hydroxyurea, (ii) VAF increase of pre-existing and/or (ii) acquisition of new mutations while on treatment correlated with higher rate of discontinuation and/or death, and (iv) reduction of *JAK2*V617F VAF associated with SVR duration.

## Introduction

Ruxolitinib is a JAK1 and JAK2 inhibitor approved for the treatment of intermediate and high-risk patients with primary (PMF) and post-polycythemia vera (PPV-MF) and post-essential thrombocythemia (PET-MF) myelofibrosis^1,2^. Long-term follow-up studies have confirmed its efficacy in inducing rapid improvements in splenomegaly, disease-associated symptomatology and overall quality of life^3^, while the impact on overall survival^3–7^ remains debated^7–9^. Furthermore, a true disease-modifying effect is questioned, since only a minority of the patients experience significant molecular responses and reduction of bone marrow fibrosis^10,11^. In spite of rapid, sometimes dramatic, clinical benefits, at least 50% of the patients become overtly refractory to ruxolitinib or experience progressively increase of spleen volume or reappearance of symptoms, necessitating soon or later discontinuation of therapy. In the COMFORT-I and COMFORT-II phase 3 trials, discontinuation due to loss of response, disease progression and treatment-related adverse events involved ≈50% of the patients at 3 years and 75% at 5 years^12–15^. Discontinuation of ruxolitinib because of loss of response was associated with dismal outcome among 107 patients enrolled in a phase 1/2 study, with median survival after discontinuation of only 14 months^16^. Managing patients who fail ruxolitinib therapy may be challenging especially when stem cell transplantation is not feasible, and options include alternative JAK inhibitor therapy, alone or in combination, in the setting of clinical trials, novel agents, splenectomy, other palliative approaches^17^.

There is yet no mechanistic explanation for the development of resistance to ruxolitinib^18^. Advocated mechanisms include reactivation of JAK/STAT signaling by JAK heterodimer formation^19^, protective effects of cytokines^20,21^, incomplete target inhibition by type I JAK inhibitor as is ruxolitinib^21^, while acquired activating *JAK2* mutations have been described in cell lines but not yet reported in patients^22,23^.

To date few studies have investigated the molecular variable that may be associated with response and durability of response to ruxolitinib. We reported that type of driver mutations and presence of HMR mutations at baseline, were indifferent as regards the obtainment of clinical responses (SVR and symptomatic improvement) at week 24 and week 48 in the COMFORT-II trial^24^. On the other hand, it was suggested that a *JAK2*V617F variant allele frequency (VAF) > 50% was associated with greater likelihood of SVR^25^. On the other hand, in a series of a long-term treated patients included in a phase 1/2 trial and analyzed by NGS panel of 28 non-driver recurrently mutated genes, the number of non-driver mutations at baseline had an impact on SVR; patients with 2 or less mutations had nine-fold higher odds of achieving SVR than those with 3 or more mutations, who also had shorter time to discontinuation of therapy^26^. A shorter time to treatment failure was also noticed in a study of 100 patients, including 23 treated with momelotinib, in association with an HMR profile and presence of *ASXL1* and *EZH2* mutations^27^. More recently, among 86 patients receiving ruxolitinib for a median of 79 months, clonal evolution, hallmarked by acquisition of new mutations under treatment, was associated with significantly shorter survival after therapy discontinuation compared to patients without clonal evolution^16^.

The purpose of our study was to analyze first, whether attainment and duration of clinical responses in patients with MF receiving ruxolitinib in a real-life setting was associated with unique mutation landscape at baseline and/or changes of mutation profile and VAF at follow-up, and whether clonal evolution might be attributed directly to selective pressure induced by ruxolitinib on pre-existing clones and/or through the facilitation of emergence of new mutated clones, in comparison with standard therapy represented by hydroxyurea.

## Materials and methods

### Patients

Seventy-one patients (42 with PMF, 29 with PPV/PET-MF) in active follow-up at our Institution were included. Diagnosis of PMF fulfilled the 2016 revised World Health Organization (WHO) criteria^28,29^ while criteria of the International Working group for Myelofibrosis Research and Treatment (IWG-MRT) were used for the diagnosis of PPV-MF and PET-MF^30^. Twenty-five patients (19 PMF, 6 PPV/PET-MF) were treated with hydroxyurea (HU) and 46 (23 PMF, 23 PPV/PET-MF) with ruxolitinib in a real-life setting. The study inclusion criteria for patients receiving ruxolitinib and HU were: (1) to have been treated continuously with the drug for at least 1 year and (2) to have stored a “baseline” blood sample (at the time of treatment start) and a “follow-up” sample collected at least one year later that, for patients who discontinued, was coincident with treatment discontinuation. Patients treated with HU were randomly selected from our database to match, in terms of diagnosis, clinical characteristics and follow-up criteria, the patients receiving ruxolitinib; all included patients had to be DIPSS intermediate-2/high risk and have a baseline spleen that was palpable at > 5 cm from the left costal margin (LCM). The dose of HU was according to clinical practice and according to the label for ruxolitinib; both drugs were titrated depending on clinical and hematologic criteria and toxicity. There was no hold of treatment in either group during the study period. All patients provided written informed consent to participate to the study, sponsored by AGIMM (AIRC- Gruppo Italiano Malattie Mieloproliferative) and supported by MYNERVA (Myeloid Neoplasms Research Venture-AIRC) project; the study was approved by local Ethical Committee.

### Definitions of clinical response and outcome

Response of symptoms or splenomegaly was according to the IWG-MRT/ELN criteria^31^. A spleen response was adjudicated in case a spleen extending 5 to 10 cm from the LCM at baseline became no palpable or a spleen that was > 10 cm decreased by ≥ 50%. A symptoms response was considered in case of a > 50% reduction of the MPN-SAF Total Symptom Score (MPN-SAF TSS)^32^. Time to clinical response and to response loss was calculated from baseline to the date of achieving or loosing, respectively, the above criteria of spleen and/or symptoms response. Overall survival was calculated from the first day of treatment to the last follow-up or death. Time to discontinuation was calculated from the date of treatment start to the date of therapy discontinuation.

## Methods

All patients were annotated for driver mutations and an additional panel of 24 “myeloid” genes by Next Generation Sequencing (NGS). Mutational analysis was performed on high-quality DNA obtained from density gradient-purified granulocytes from peripheral blood. Samples were collected before starting HU/ruxolitinib therapy (baseline) and at different time points thereafter; the last available sample (“follow-up” -FU- sample for the purpose of this study) was collected at least one year later for patients who were still receiving the drug (in case of patients with sustained response), or at therapy discontinuation for patients who discontinued because of no response/loss of response.

*JAK2*V617F and *MPL* W515 L/K mutations were detected by real time (RT)-qPCR^33,34^ and high-resolution melting analysis (HRMA)^35^ followed by bidirectional Sanger sequencing^36^, respectively. *CALR* mutations were identified by bidirectional sequencing and capillary electrophoresis (CE) and classified as type 1/like or type 2/like, as described^37,38^. *CALR* amplification was carried out with a 6-FAM-labeled forward primer followed by fragment analysis on a ABI Prism 310 Genetic Analyser (GeneMapper Software 4.1; Applied Biosystems, Forest City, CA, USA). All samples with an additional peak to the wild-type one were further evaluated by direct Sanger sequencing. The level of detection was < 0.1% for *JAK2*V617F mutations and 1% for *MPL*W515 mutations using RT-qPCR and HRMA, respectively, and 1% by capillary electrophoresis for *CALR* mutations.

High molecular risk mutations (HMR; *ASXL1*, *EZH2*, *SRSF2*, *IDH1*, *IDH2*) and other myeloid-neoplasm associated gene mutations (*CBL*, *C-KIT*, *CSF3R*, *CUX1*, *DNMT3A*, *ETNK1*, *IKZF1*, *KRAS*, *NFE2*, *NRAS*, *PTPN11*, *RUNX1*, *SETBP1*, *SH2B3*, *SF3B1*, *TET2*, *TP53*, *U2AF1*, *ZRSR2*), as well as the entire coding regions of JAK2 and MPL, were evaluated by using a custom panel for NGS on Ion Torrent PGM platform (ThermoFisher Scientific, Waltham, Massachussets, USA). NGS raw reads were aligned against the GRCh38/hg38 using NextGENe® software 2.4.2 for variants call with a variant allele frequency (VAF) threshold of ≥ 2%, in case of previously unreported mutations, and ≥ 1% for known hotspots, and depth of coverage of at least 100 × (SoftGenetics, LLC, State College, PA). Mutations in the exonic regions were filtered by available databases (dbSNP, COSMIC, 1000 genome, ExAC); protein function predictor algorithms (Polyphen2, SIFT, MutationTester, FATHMM, Gerp) were used to predict functional relevance of mutations, and only indels and pathogenetic variants were considered. An HMR category was defined by the presence of ≥ 1 of HMR genes mutations; patients lacking these mutations were defined at “low molecular risk” (LMR). For the analysis, we recorded any modification of VAF, either decreasing or increasing, that had a magnitude of at least 20% compared to baseline. Mutations were defined “acquired” in case of de novo detection ( ≥ 2% VAF) and “lost” in case the VAF of a mutation detected became less than 0.1%.

### Statistical analysis

Best overall response of splenomegaly was adjudicated at any time while the patients was on continuous drug administration using the IWG-MRT/ELN revised criteria^31^; assessment at 24 and 48 weeks was also performed, as in the COMFORT trials. Response of symptoms was annotated at any time point between baseline and 48 weeks. Categorical variables were compared using χ2 or Fisher’s exact test and continuous variables were compared using Mann-Whitney or Kruskal-Wallis tests. Comparison of response to ruxolitinib across molecular categories was tested for homogeneity of distributions using chi-square test. Survival time estimates, including survival curves by response status, were obtained with the Kaplan-Meier method; the hazard ratio (HR) was determined using a Cox proportional hazards model. All *P* values are 2-tailed and were considered significant when *P* < .05. Statistical analyses were performed using SPSS v.25 (IBM).

## Results

### Patients’ characteristics at baseline

Patients’ characteristics at baseline are reported in Table 1; forty-six patients received ruxolitinib (ruxo-patients) and 25 patients received hydroxyurea (HU-patients). There was no statistically significant difference between the two groups regarding main clinical and laboratory parameters (Table 1), a part for a greater proportion of patients with larger ( > 10 cm from LCM) splenomegaly among the ruxo-patients compared to HU-patients (76.1% vs. 40.0%; *P* < .0001). The median follow-up duration from the initiation of therapy was 3.4 years (range, 1.0–7.4; *P* = .981) in patients receiving ruxolitinib and 2.9 years (range, 1.0–11.1) in HU-patients. The proportion of study patients who had a follow-up sample collected at > 5 years of treatment was 36.0% and 34.8% for ruxolitinib and HU, respectively (*P* = .897). Over the study period, 11 of 46 (23.9%%) ruxo-patients discontinued therapy for loss of response, 1 patient (2.2%) progressed to acute leukemia and 20 patients (43.5%) died; fifteen patients (32.6%) were still receiving ruxolitinib at latest follow-up. In the HU group, 10 patients (40.0%) died during the follow-up, one (4.0%) of which had progressed to acute leukemia, while 15 (60%) were still on therapy.

**Table 1:** Baseline clinical and hematologic characteristics of study patients stratified according to the treatment

Variables	Ruxo-patients (*N* = 46)	HU-patients (*N* = 25)	*P*
Diagnosis, *n*.(%)			
PMF	23 (50.0)	19 (76.0)	.050
PPV-MF	16 (35.0)	2 (8.0)	
PET-MF	7 (15.0)	4 (16.0)	
Follow-up from the start of treatment, *y*: median (range)	3.4 (1.0–7.4)	2.9 (1.0–11.1)	.981
Time from diagnosis to treatment	1.1 (0.3.6)	2.8 (0–3.7)	.743
Males, *n* (%)	22 (48.0)	15 (60.0)	.232
Age, y; median (range)	63.4 (35.0–81.0)	66.9 (43.0–88.0)	.087
Hemoglobin, g/L; median (range)	107 (70–140)	99 (89–109)	.912
Leukocytes, x10^9^/L; median (range)	13.3 (3.0–103.0)	11.8 (5.8–14.0)	.917
Platelets, x10^9^/L; median (range)	333 (52–750)	433 (227–1378)	.090
Circulating blasts ≥ 1%; *n* (%)	12 (26.1)	7 (28.0)	.993
Constitutional symptoms; *n* (%)	43 (93.5)	21 (84.0)	.558
Splenomegaly > 10 cm from LCM; *n* (%)	35 (76.1)	10 (40.0)	<.0001
Patients with cytogenetic information; *N* = (% of total)	42 (91.3)	21 (84.0)	.202
Abnormal cytogenetics	19 (45.2)	6 (28.6)	
Unfavorable karyotype	4 (9.5)	2 (9.5)	
DIPSS			
Intermediate-2	40 (86.9)	20 (80.0)	.441
High	6 (13.1)	5 (20.0)	

*Note*: Unfavorable karyotype indicates any of the following: + 8, –7/7q–, i(17q), inv(3), –5/5q, 12p–, or 11q23 rearrangements. DIPSS, Dynamic International Prognostic Scoring System. DIPSS uses five independent predictors of inferior survival: age > 65 years, hemoglobin < 10 g/dL, leukocytes > 25 × 10^9^/L, circulating blasts ≥ 1%, constitutional symptoms, resulting in four (low, intermediate-1, intermediate-2 and high) risk categories

### Mutation landscape at baseline

**Fig. 1:**
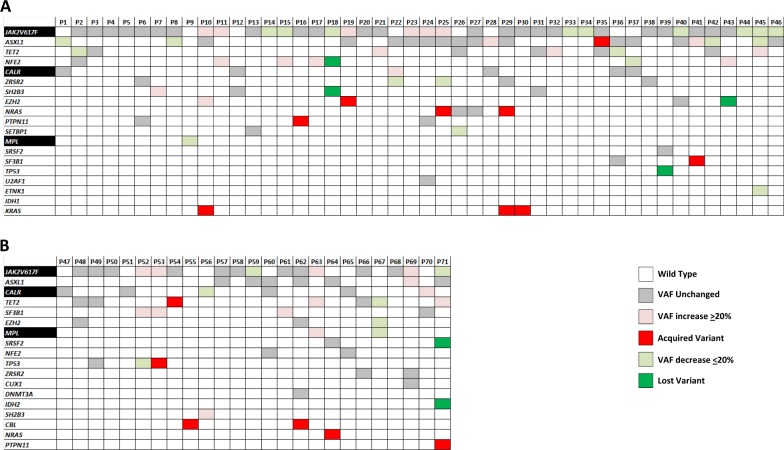
Landscape plot of mutations in the study population. Each column represents an individual patient. **a**: ruxolitinib treated patients; **b**: HU-treated patients. color code: gray indicates a mutation detected at baseline that remained unchanged at the latest follow-up sample; pink colour indicates a mutation whose VAF increased of at least 20% compared to baseline; red colour indicates a newly acquired mutation at follow-up; light green colour indicates a mutation whose VAF decreased of at least 20% compared to baseline; green colour indicates a mutation that, while detected at baseline, was no longer detected in the follow-up sample

The mutation landscape of patients at baseline is shown in Fig. 1. A *JAK2*V617F mutation was found in 54 patients (76.0% of total; 82.6% of ruxo-patients and 64% of HU-patients), *CALR* mutation in 12 (16.9%; 13% of ruxo-patients and 24% of HU-patients), of which 10 (83%) were Type 1 and 2 (17%) Type 2, *MPL* mutations in 3 patients (1 in the ruxo-group and 2 in the HU-group); 1 patient in each cohort was triple negative. Non-driver mutations represented at > 5% in the series were *ASXL1* (36.6%; 41.3% of ruxo-patients and 28.0% of HU-patients), *TET2* (22.5%; 21.7% of ruxo-patients and 24.0% of HU-patients), *NFE2* (12.6; 15.2% of ruxo-patients and 8.0% of HU-patients), *ZRSR2* (11.7%; 11.6% of ruxo-patients and 11.8% of HU-patients), *EZH2* (8.5%; 6.5% of ruxo-patients and 12.0% of HU-patients), *SF3B1* (7.0%; 2.2% of ruxo-patients and 16.0% of HU-patients), *SH2B3* (7.0%; 8.7% of ruxo-patients and 4.0% of HU-patients). Mutations of *TP53* were found in 3 patients (4.2%), 2 of whom were in the HU-group. No mutation was found in *CBL, C-KIT, CSF3R, CUX1, DNMT3A, IKZF1, RUNX1, IDH2*. Considering all non-driver mutated genes evaluated, a total of 54 patients (76%) showed at least one somatic variant: 36 in the ruxo-group (78.3%) and 18 (72.0%) in the HU-group; 2 or more mutations were found in 19 ruxo-patients (41.3%) and 9 HU-patients (36.0%); 3 or more mutations were harbored by 4 ruxo-patients (8.7%) and 2 HU-patients (8.0%). A total of 30 patients (42.2%) were considered at high-molecular risk (HMR), including 21 in the ruxo-group (45.6%) and 9 (36.0%) in the HU group; 2 or more HMR mutations were found in 5 patients (7.0%), of which 2 in the ruxo-group (8.0%) and 3 in HU-patients (12%). With the limitation of small numbers, we compared rate of non-driver mutations in patients with PMF vs. PPV/PET MF. A total of 78.6% of PMF vs. 72% of PPV/PET MF patients had ≥ 1 mutation (*p* = 0.58), and 40.5% of PMF vs. 44.8% of PPV/PET-MF were HMR^+^.

### Mutation landscape at follow-up sample

**Fig. 2:**
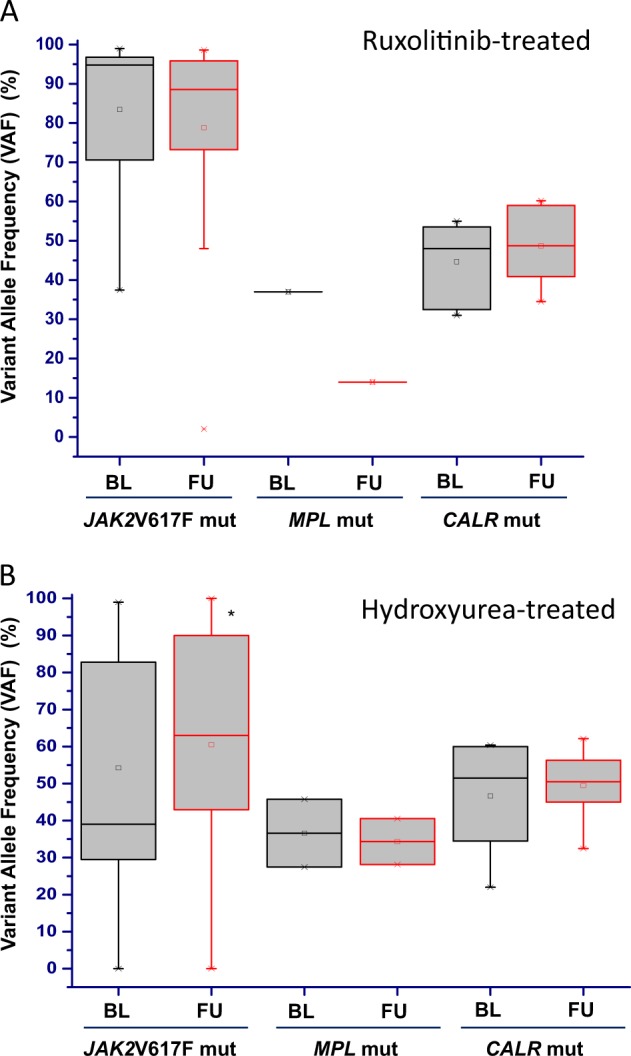
Changes of driver mutation variant allele frequency (VAF) over study period. The VAF of the *JAK2*V617F, *MPL*W515x and *CALR* driver mutation was measured in samples collected at baseline (BL) and at the latest available follow-up (FU) in patients receiving ruxolitinib (**a**) and hydroxyurea (**b**)

In the follow-up sample of patients receiving ruxolitinib, a change, either increase or decrease (see Materials and Methods for definition) of the VAF of any mutation (driver and non-driver) detected at baseline was detected in 41.3% and 34.8%, respectively (Fig. 1, panel A). Concerning driver mutations (Fig. 2, panel A), the *JAK2*V617F VAF overall decreased from 83.4 ± 19.3% to 78.8 ± 25.3% (*P* = .43): in detail, the VAF increased in 6 patients (15%; median increase + 27%, range + 20% to + 50%) and decreased in 9 (23%; median −39%, range −21 to −62%); *CALR* VAF showed a slight, not significant increase from 44.7 ± 10.6% to 48.7 ± 10.3% (*P* = .52): in detail it increased by 32% in 1 patient and remained unchanged in 5 patients; in the one *MPL* mutated patient, the VAF decreased from 37 to 14% (−38%). As regards non-driver mutations (Fig. 1, panel A), the VAF overall increased in the follow-up sample in 11 patients (24% of all mutated patients) by a median value of 104%, ranging from + 20% to + 480%, compared to baseline one. The involved genes were *NFE2* (number of variants = 4), *TET2* (number of variants = 3), *ASXL1* (number of variants = 2), *SH2B3* and *EZH2* (one variant each). On the contrary, the VAF decreased in 10 patients (22%) by a median of 30% (−23% to −69%); the involved genes were *ASXL1* (number of variants = 4), *TET2* and *ZRSR2* (2 variants each), *NFE2*, *SETBP1*, and *ETNK1* (1 variant each). Four mutations detected at baseline in 3 patients (6.5%; Fig. 1, panel A), involving *EZH2, NFE2, SH2B3*, and *TP53*, were no longer detected in the follow-up sample. Conversely, acquisition of a new mutation was observed in 8 cases (17.4%; Fig. 1, panel A), involving *EZH2*, *SF3B1*, *ASXL1*, *PTPN11* (one each), *NRAS* (2 cases), and *KRAS* (3 cases), in one case concurrently with acquisition of a *NRAS* mutation. Of note, acquisition of novel mutations was observed only in *JAK2*V617F mutated patients compared to no *CALR* mutated patients.

Among patients receiving HU, an increase or a decrease of the VAF of any baseline mutation (driver and non-driver) was detected in 20.8% and 33.3%, respectively (Fig. 1, panel B). Concerning the driver mutations (Fig. 2, panel B), the *JAK2*V617F VAF overall increased from 54.2 ± 20.3% to 60.5 ± 22.7% (P = .59), in detail, the VAF increased in 4 patients (16%, range + 33% to + 88%) and decreased in 2 patients by 41% and 63%, respectively. In *CALR* and *MPL* mutated patients the VAF increased in 1 case and decreased in 1 case each (Fig. 2, panel B). As regards non-driver mutation (Fig. 1, panel B), the VAF overall increased in 7 patients (28% of all mutated patients) by a median + 94.9% (range + 22.8% to + 174%) in the follow-up sample compared to baseline one; the involved genes were *SF3B1* and *TET2* (number of variants = 2), *ASXL1* and *SH2B3*, (1 variant each). On the contrary, the VAF decreased in 2 patients (8%), the genes involved were *TP53* (−65%) and *TET2* (−50%). One patient showed disappearance of baseline mutation in *SRSF2* and *IDH1* (VAF of 6.3% and 8.2%, respectively), while acquisition of new mutation at follow-up sample occurred in 6 patients (24%); involved genes were *TET2*, *TP53*, *NRAS*, *PTPN11* and *CBL* (in 2 patients) (Fig. 1, panel B). Acquisition of novel mutation was observed in four *JAK2*V617F mutated patients and in 2 triple negative patients, compared to no patients with *CALR* mutation.

Clonal progression occurred in 21.4% of PMF vs. 17.2% of PPV/PET MF (*P* = .66).

### Correlation of mutation landscape at baseline with clinical response

Among patients receiving ruxolitinib, response of symptoms and splenomegaly (by IWG-MRT/ELN criteria^31^) was achieved by 78 and 57% of the patients after a median of 2.0 months (range, 1–37 months) and 5.8 months, respectively (range, 1–49 months); of these, 11 (31%) and 12 (30.8%) patients lost clinical response after a median of 2.4 years (range, 0.5–5.2) for symptoms and 1.8 years for SVR (range, 0.7–5.0). Since in the HU group only 1 (4%) and 3 (12%) patients had achieved symptoms and spleen response, respectively, at any time during follow-up, further analysis was restricted to patients treated with ruxolitinib.

We found no difference in the proportion of patients with *JAK2*V617F mutation vs. patients with *CALR* mutation who achieved either a spleen or symptom response, confirming previous reports; a SVR and/or a symptom response was obtained by 54.1% and 86.0% of *JAK2*V617F mutated patients compared to 66.7% and 60.0% of *CALR* mutated patients. There was also no difference in terms of SVR and/or a symptom response among patients with a *JAK2*V617F VAF greater or lower than 50%.

**Fig. 3:**
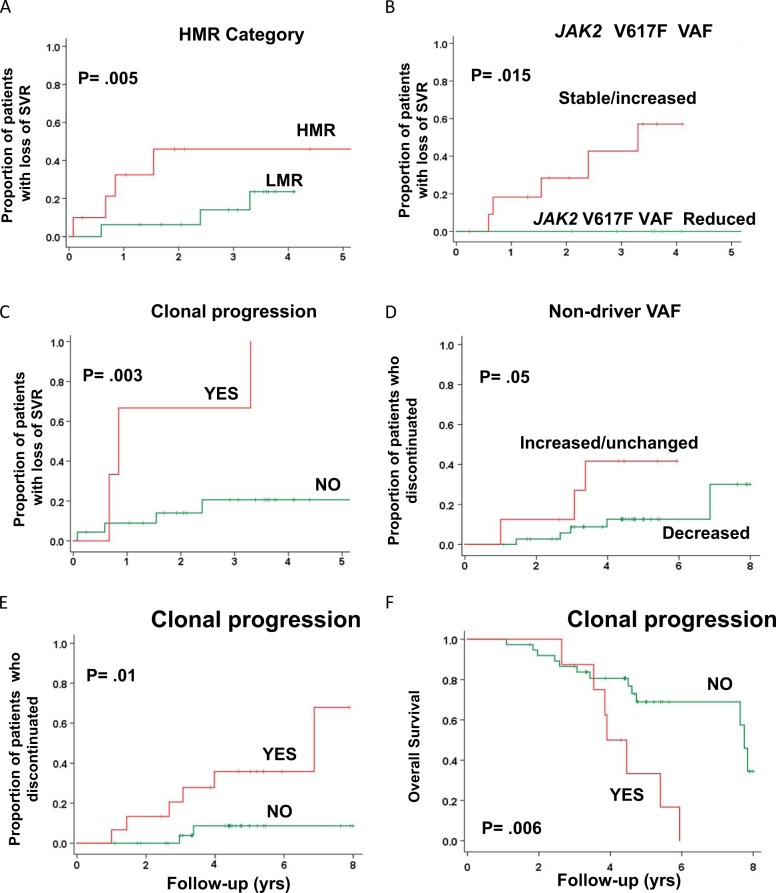
Correlation of mutation profile, at baseline and during follow-up, with maintenance of spleen volume response, treatment duration and outcome in patients receiving ruxolitinib. Kaplan-Meyer estimates of the proportion of patients treated with ruxolitinib who presented loss of spleen response, according to IWG-MRT criteria, are shown in panels **a**–**d** in relation to: a HMR status at baseline (**a**); modifications of *JAK2* V617F VAF at latest follow-up compared to baseline (**b**); clonal progression, considered as the appearance of a novel somatic variant in the follow-up sample (**c**); modifications of the VAF of any non-driver mutation al follow-up sample compared to baseline (**d**). Kaplan-Meyer estimates of the proportion of patients who discontinued ruxolitinib in relation to acquisition of clonal progression at follow-up sample are shown in **e**. **f** shows Kaplan-Meyer estimates of overall survival, measured from therapy initiation in patients treated with ruxolitinib depending on the acquisition of clonal progression in the follow-up sample

Presence of an HMR status at baseline did not affect the likelihood of obtaining SVR or symptom response, as we previously reported^24^. A spleen response was obtained by 42.9% of HMR patients compared to 44.0% of the LMR counterpart at 24 weeks, and 42.8% compared to 56.0% at 48 weeks; the best overall spleen response rate was 51% and 64%, respectively. The rate of symptoms response was 68.4% in HMR patients compared to 64.0% in un-mutated patients at 24 weeks, and 73.7% compared to 76.0% at 48 weeks. On the other hand, baseline HMR status was significantly associated with loss of SVR, with a HR of 3.6 (95%CI, 12.0–16.7; *P* = .005) compared to LMR patients (Fig. 3, panel A); at 3 years, 14% of LMR patients had lost spleen response compared to 46% of the HMR category (*P* = .005). There was no difference in rate of symptom response depending on HMR vs. LMR status.

We also analyzed the impact of sole *ASXL1* mutations at baseline, the most frequently mutated gene, on response. Presence of *ASXL1* mutation at baseline did not impact on the rate of spleen or symptom response. A spleen response was obtained by 47.4% of *ASXL1*- mutated patients compared to 40.7% of the un-mutated counterpart at 24 weeks, and 47.0% compared to 51.9% at 48 weeks. Symptoms response was achieved by 64.7% of *ASXL1* mutated patients compared to 66.7% at 24 weeks, and 70.6% compared to 77.8% at 48 weeks. Furthermore, presence of sole *ASXL1* mutation was associated with a significantly shorter duration of spleen volume reduction; the probability of maintaining a spleen response at 3 years among *ASXL1* mutated patients was 33% compared to 87% of un-mutated patients (*P* = .009).

### Impact of clonal evolution at follow-up on clinical response

A decrease of the *JAK2*V617F VAF at any time point during treatment was significantly associated with maintenance of SVR (*P* = .015); in fact, none of the 7 patients who showed decrease of ≥ 20% from baseline *JAK2*V617F VAF lost SVR compared to 6 out of 13 (46.1%) who showed stable or increased *JAK2*V617F VAF (HR = 61.8, 95% CI 1.01–870.2; Fig. 3, panel B). Similar analysis could not be done for *CALR* mutated patients, owing that there was no significant modification of *CALR* VAF during follow-up.

We found that loss of SVR was significantly associated with clonal progression, *ie* the acquisition of ≥ 1 novel mutation in any non-driver genes during follow-up; all patients with SVR loss during follow-up had evidence of clonal progression compared to 21% who lost SVR without having clonal progression (*P* = .006). Patients with clonal progression had median duration of SVR of 10 months (range, 8.4–13.0 month) compared to not-reached in patients without clonal progression (HR 7.2, 95% CI, 1.6–33.0; *P* = .003) (Fig. 3, panel C). A greater rate of therapy discontinuation due to SVR loss was found in patients showing VAF increase of any baseline mutation; at 4 years of treatment, the proportion of patients who discontinued ruxolitinib was 36% among those with VAF increase compared to 9% in those where VAF remained stable or decreased (HR 6.1, 95% CI 1.2–30.5; *P* = .01; Fig. 3, panel D). Clonal progression was also associated with a higher rate of treatment discontinuation (38% vs. 13% for those without clonal progression; *P* = .05), accounting for an HR of 3.9 (95%CI, 1.1–17.3; *P* = .01) (Fig. 3 panel E).

We did not find any impact of modifications of VAF of *JAK2*V617F and other non-driver mutations on survival whilst acquisition of clonal progression at any study time point was associated with shorter overall survival; the median OS was 3.9 years (range, 3.1–4.6) compared to 7.7 years (7.5–8.0) for patients without clonal progression (HR = 3.6, 95%CI 1.4–9.7; *P* = .006; Fig. 3 panel F). As many as 87.5% of patients with clonal progression documented at any study time point died compared to 34% of those without clonal progression (*P* = .006).

## Discussion

Results of current study confirm and extend previous reports on the impact of driver^39,40^ and non-driver^24,26,27^ baseline mutations, and of mutations acquired while on treatment (clonal progression)^16^, on response to treatment and response duration in patients with myelofibrosis receiving ruxolitinib. For the first time, our study included a control group of matched patients treated with hydroxyurea, that highlighted that MF is hallmarked by highly dynamic mutation landscape that is largely treatment-independent, since modifications of mutation profile during follow-up were substantially similar in patients receiving ruxolitinib or hydroxyurea. These findings, while indicating that clonal progression is not facilitated by ruxolitinib itself but is intrinsically associated to the disease, may have practical relevance as concerns safety issues of ruxolitinib, particularly in the light of recent demonstration of aggressive B-cell lymphomas developing in ruxo-treated patients, that were shown to stem from B-cell clones pre-existing in the bone marrow^41^. We acknowledge that one limitation of the study is the limited number of patients in the hydroxyurea group, that might warrant confirmation in larger series. Of note, progression to PPV-MF and to acute leukemia in PV and MF patients receiving ruxolitinib was not increased compared to controls^3,4,14^. However, acquisition of new mutations while on ruxolitinib has relevant clinical correlates, since it was associated with higher rate of discontinuation due to resistance to treatment and death.

Ruxolitinib has limited effects on *JAK2*V617F VAF in both MF^10,15^ and PV^42^, although some patients may present sustained decrease, irrespective of initial level^10^; complete molecular remissions are exceptional^43^. Short-term SVR occurs independent of changes in *JAK2*V617F VAF^15^; however, we report the novel observation that patients with *JAK2*V617F VAF reductions ≥ 20% from baseline have significantly greater likelihood to maintain sustained SVR. On the other hand, unlike previous report^25^, we observed no impact of baseline *JAK2*V617F VAF on SVR. An additional finding, that deserves validation in larger series, was that clonal progression virtually segregated with patients harboring *JAK2*V617F mutation, and was not observed in *CALR* mutated patients. This may have to do with the increased genomic instability that characterizes cells expressing mutated JAK2^44^, showing enhanced frequency of spontaneous homologous recombination events, DNA double strand breaks^45^, high levels of NHE-1^46^ and deamidate Bcl-x_L_^47^, that are possibly mediated by increased reactive oxygen species (ROS)^48,49^.

Overall, findings from this study add novel information on the impact of baseline and on-treatment acquired genomic alterations on the clinical response in patients with MF receiving ruxolitinib, further highlighting the complexity of genomic landscape of these patients. The ultimate goal of this kind of studies is to identify a molecular profile at baseline that might guide decision regarding initiation of therapy with ruxolitinib;^50^ although presence of some molecular assets at baseline argue against a long-term likelihood to maintain SVR (current study and^16,24,26,27^), the large majority of patients obtain rapid clinical improvements, that are not otherwise achieved with other agents, making use of ruxolitinib a reasonable upfront strategy in symptomatic patients. Conversely, detection of specific molecular assets might be useful in counseling for an earlier shift from ruxolitinib to experimental therapies and/or stem cell transplantation. Probably the most relevant variable impacting on long-term maintenance of SVR to ruxolitinib, as well as on survival in patients who discontinue^16^, is the development of clonal progression while on treatment; serial assessment of mutation profile would be required to define whether the individual has manifested or not clonal progression, that is practically cumbersome^50^. All together, these particular observations might advocate for the prospective use of extensive genotyping in patients receiving ruxolitinib; however, we believe that further information are needed before this approach may be recommended in daily practice, also considering that alternative therapeutic options to ruxolitinib, outside clinical trials, in non-transplant eligible patients are very few and largely unsatisfactory^17^.
